# The next chapter in eating disorder prevention? Findings from a randomized controlled trial of a mindfulness-based intervention aimed at reducing risk for disordered eating

**DOI:** 10.1186/2050-2974-1-S1-O34

**Published:** 2013-11-14

**Authors:** Melissa Atkinson, Tracey Wade

**Affiliations:** 1 Flinders University, Australia

## 

The primary objective of this study was to evaluate a mindfulness-based prevention program against an established dissonance program with regard to reducing risk for disordered eating. Adolescent girls (N = 379, Mean age = 15.70, SD = 0.77) from four high schools were randomly allocated by class to receive either a mindfulness or dissonance-based program, delivered universally, or lessons as normal (assessment-only control). Standardised measures of eating disorder behaviours and related risk factors were completed at baseline, post-program, 1-month and 6-month follow-up. Controlling for baseline, results showed significant group differences over time for weight concerns (F (6, 663) = 3.74, p = .001), with both mindfulness and dissonance groups showing a greater reduction than control. No other variables demonstrated significant interactions, however main effects of time for dietary restraint, mindfulness, self-compassion, emotion dysregulation, negative affect, escape-avoidant coping, and media internalisation indicated overall improvement across groups. Improvements evident within the control group may suggest the presence of cross-contamination between classes or an impact of assessment. Further analysis is required to account for significant missing data across time points; however, these preliminary findings validate continued evaluation of mindfulness in this context.

This abstract was presented in the **Prevention** stream of the 2013 ANZAED Conference.

